# Identification of a thirteen-gene signature predicting overall survival for hepatocellular carcinoma

**DOI:** 10.1042/BSR20202870

**Published:** 2021-04-22

**Authors:** Xiaohan Zhou, Chengdong Liu, Hanyi Zeng, Dehua Wu, Li Liu

**Affiliations:** 1 Department of Radiation Oncology, Nanfang Hospital, Southern Medical University, Guangzhou 510515, Guangdong Province, People’s Republic of China; 2 Department of Infectious Diseases, Nanfang Hospital, Southern Medical University, Guangzhou 510515, Guangdong Province, People’s Republic of China

**Keywords:** Gene signature, HCC, Infiltration of immune cells, Risk-score model, Tumor microenvironment

## Abstract

**Background:** Hepatocellular carcinoma (HCC) is a malignant tumor of the digestive system characterized by mortality rate and poor prognosis. To indicate the prognosis of HCC patients, lots of genes have been screened as prognostic indicators. However, the predictive efficiency of single gene is not enough. Therefore, it is essential to identify a risk-score model based on gene signature to elevate predictive efficiency.

**Methods:** Lasso regression analysis followed by univariate Cox regression was employed to establish a risk-score model for HCC prognosis prediction based on The Cancer Genome Atlas (TCGA) dataset and Gene Expression Omnibus (GEO) dataset GSE14520. R package ‘clusterProfiler’ was used to conduct function and pathway enrichment analysis. The infiltration level of various immune and stromal cells in the tumor microenvironment (TME) were evaluated by single-sample GSEA (ssGSEA) of R package ‘GSVA’.

**Results:** This prognostic model is an independent prognostic factor for predicting the prognosis of HCC patients and can be more effective by combining with clinical data through the construction of nomogram model. Further analysis showed patients in high-risk group possess more complex TME and immune cell composition.

**Conclusions:** Taken together, our research suggests the thirteen-gene signature to possess potential prognostic value for HCC patients and provide new information for immunological research and treatment in HCC.

## Introduction

Hepatocellular carcinoma (HCC) is a common malignant tumor of human digestive system. Its early diagnosis and treatment are becoming a worldwide problem [1]. Therefore, seeking prognostic markers is urgent for identifying high-risk patients of HCC patients, which may be helpful to implement individualized treatment.

Increasing studies have shown that gene signatures have an advantage in predicting the overall survival (OS). Long et al. constructed a four-gene signature based on CENPA, SPP1, MAGEB6 and HOXD9 expression to predict the OS of HCC [2]. Zheng et al. also established a four-gene signature based on SPINK1, TXNRD1, LCAT and PZP to predict the OS of HCC patients in The Cancer Genome Atlas (TCGA) cohort [3]. However, little research has been done to study causes of different risk between patients, such as the composition of tumor microenvironment (TME).

In our study, we constructed a thirteen-gene signature-based risk-score model that included LDHA, GPC1, GRM8, PPAT, SLC29A3, EMCN, GDI2, CBX2, LILRA2, ADAMTS5, GSR, WEE1 and SLC1A5. The risk-score model was validated in Gene Expression Omnibus (GEO) database. Furthermore, we built and validated a nomogram in the TCGA cohort to predict OS of HCC patients. Functional analysis of the differentially expressed genes (DEGs) between high- and low-risk groups was conducted. The infiltration level of various immune and stromal cells in the TME was evaluated. In general, the prognostic model might help effectively predict OS of HCC patients and evaluate the infiltration level of immune and stromal cells in HCC tissue.

## Materials and methods

### Data preparation

TCGA and GEO database were used to establish and validate risk score model. For TCGA cohort, 371 patients possessed RNA seq data, in which 365 patients with OS data were selected for the construction of prognostic model. The TCGA mRNA expression was generated with HTseq-FPKM. Then, 365 patients were randomly grouped into 183 cases as internal training set and 182 cases as internal testing set. For external testing set, we obtained GEO HCC dataset (access no: GSE14520, platform: GPL571) which included 247 HCC samples, in which 221 patients possessed available OS information for validation of prognostic model. The clinical baseline data of TCGA and GSE14520 are provided in Supplementary Table S1.

### Construction of the risk-score prognostic model

Lasso regression analysis followed by univariate Cox regression was conducted to investigate and narrow OS related genes. A total of 436 genes that meet the standard of *P*-value <0.001 by univariate Cox regression analysis were selected for Lasso-penalized regression. Lasso-penalized regression was conducted by R package ‘glmnet’ with ten-fold cross-validation. According to the expression of each gene, we screened eligible genes and calculated corresponding coefficients and risk score for each patient. A thirteen-gene signature was established based on univariate and Lasso regression analyses. The prognosis index (PI) = (0.094 * LDHA expression) + (0.076 * of GPC1 expression) + (0.087 * GRM8 expression) + (0.279 * PPAT expression) + (0.189 * SLC29A3 expression) + (−0.147 * EMCN expression) + (0.016 * GDI2 expression) + (0.187 * CBX2 expression) + (0.637* LILRA2 expression) + (0.368 * ADAMTS5 expression) + (0.056 * GSR expression) + (0.028 * WEE1 expression) + (0.008 * SLC1A5 expression). Patients of TCGA HCC cohort were grouped into high- and low-risk groups by the optimal cut-off value. In addition, Kaplan–Meier survival and receiver operating characteristic (ROC) analysis were conducted to estimate the predictive efficiency of the risk-score model.

### Construction and validation of a predictive nomogram

To evaluate the probability of 5-year OS, we constructed a nomogram in TCGA HCC cohort [4] based on multivariable Cox regression analysis of risk-score model and clinical characteristic parameters. The calibration curve was used to show the nomogram prediction probabilities against the observed rates. We used DCA plot to calculate the clinical net benefit and net reduction. The best model possessed the highest net benefit and net reduction as calculated [5].

### DEG screening

Median risk score was used to group the patients into the high- and low-risk groups. The DEGs were selected by the R package ‘Limma’. DEGs with an absolute log2FC (fold change) > 0.5 and the *P*<0.05 were selected for subsequent analysis.

### Enrichment analyses

Gene Ontology (GO) and Kyoto Encyclopedia of Genes and Genomes (KEGG) analyses were conducted using R package ‘clusterProfiler’ [6]. Gene Set Enrichment Analysis (GSEA) was conducted using gseGO, gseKEGG and gsePathway function in R package ‘clusterProfiler’, with the parameters: nPerm = 1000, minGSSize = 10, maxGSSize = 1000, pvalue-Cutoff = 0.05.

### Generation of TME gene signatures and signature score computation

We used TME-related gene sets, the scoring method curated by Mariathasan et al. [7]. Briefly, a principal component analysis (PCA) was performed, and principal component 1 was considered to serve as gene signature score.

### Immune cell infiltration

The single-sample GSEA (ssGSEA) was used to evaluate the infiltration level of 28 immune cells. The gene sets of various immune cells were obtained from a recent publication [8]. The infiltration score of each immune cell was calculated through ssGSEA of ‘GSVA’ R package in the R software. The ssGSEA score was normalized to uniform distribution, for which the ssGSEA score is distributed between 0 and 1.

### Human tissue samples

The experiments involving human samples in our study were in accordance with the principles of the Declaration of Helsinki, and approved by the Institutional Review Board of Nanfang Hospital, Southern Medical University of Guangdong, China (NFEC-201208-K3). A total of ten liver cancer and paired non-cancerous tissues were collected and used for qRT-PCR.

### qRT-PCR

TRIzol® reagent (TaKaRa, Tokyo, Japan), PrimeScript™ First Strand cDNA Synthesis Kit (TaKaRa, Tokyo, Japan) and SYBR® Green PCR kit (TaKaRa, Tokyo, Japan) were used to perform the extraction of total RNA, synthesis of First-Strand cDNA and real-time PCR, respectively. Primers are provided in Supplementary Table S2.

### Statistical analysis

All data are presented as the mean ± standard deviation (SD). Student’s *t* test (two-tailed) was used to analyze differences between two groups using R software (version: 3.6.2). *P*<0.05 was considered statistically significant: *P<0.05; **P<0.01; ***P<0.001 and ****P<0.0001.

## Results

### Risk-score model construction

**Figure 1:**
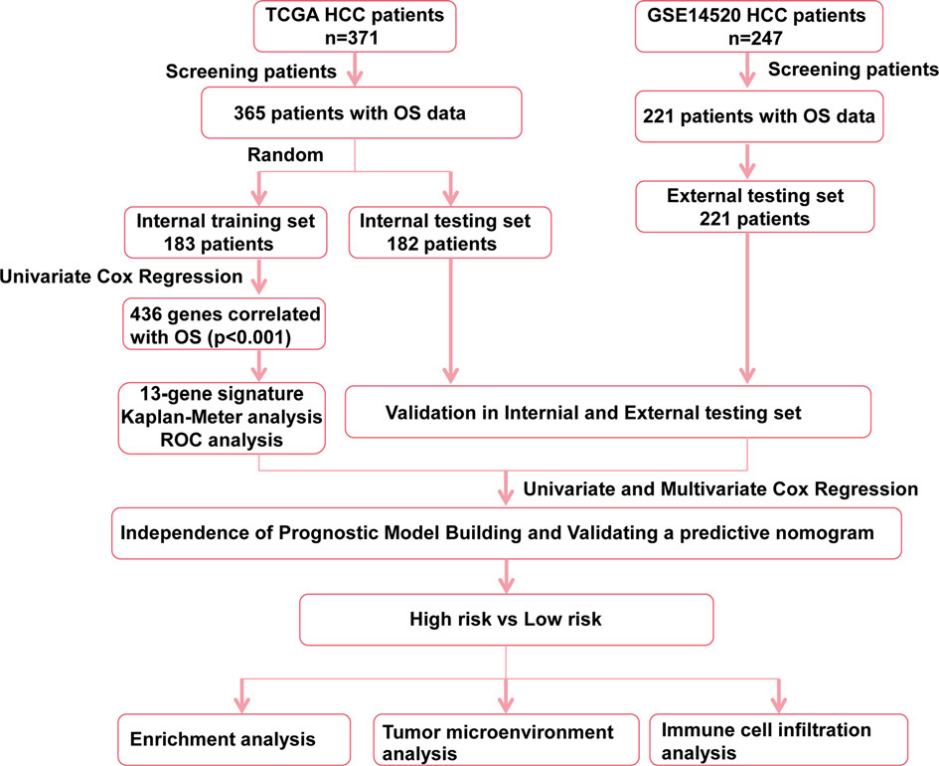
The work flow showing the scheme of our study

**Figure 2:**
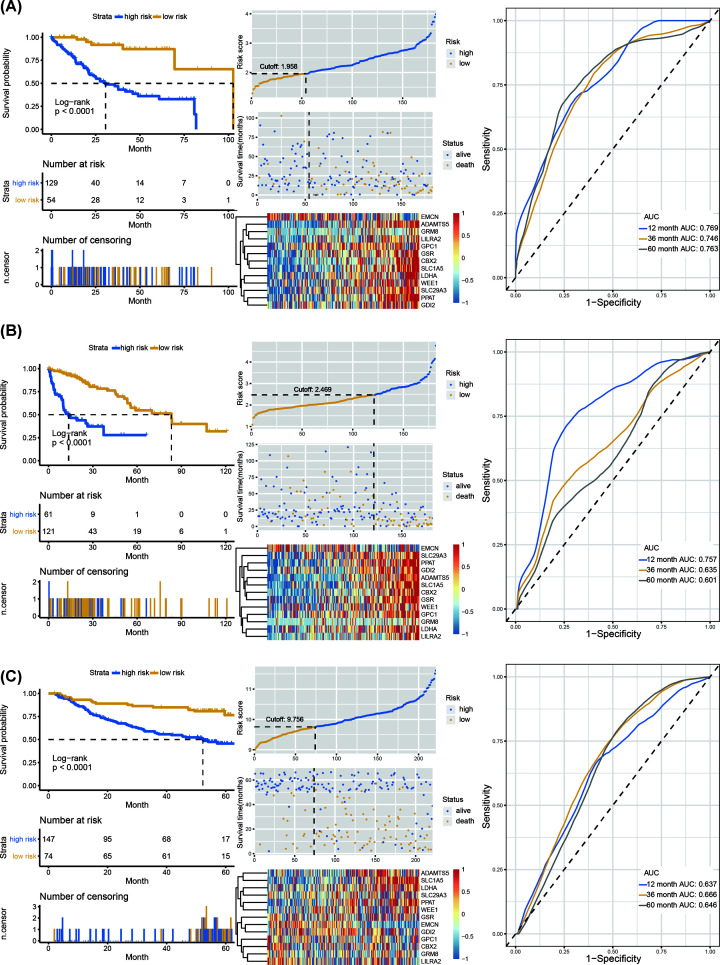
Construction of risk-score predictive model (**A**) Kaplan–Meier curve, heatmap of normalized mRNA expression, scatterplot of risk score and patient survival status, and ROC curve of the risk-score model in the internal training set of TCGA cohort. (**B**) Kaplan–Meier curve, heatmap of normalized mRNA expression, scatterplot of risk score and patient survival status, and ROC curve of the risk-score model in the internal testing set of TCGA cohort. (**C**) Kaplan–Meier curve, heatmap of normalized mRNA expression, scatterplot of risk score and patient survival status, and ROC curve of the risk-score model in the external testing set of GSE14520 cohort.

Three hundred and sixty-five HCC patients in TCGA cohort were included. These patients were randomly separated into two groups, including internal training set (*n*=183) and internal testing set (*n*=182). Univariate Cox regression analysis identified 436 OS-related genes (*P*<0.001) (Supplementary Table S3). Then, Lasso analysis was conducted in the internal training set to further shrink the OS-related genes. Thirteen genes (including LDHA, GPC1, GRM8, PPAT, SLC29A3, EMCN, GDI2, CBX2, LILRA2, ADAMTS5, GSR, WEE1 and SLC1A5) were screened and subsequently used to construct a prognostic risk-score model (Figure 1). We further calculated the risk score for each patient and seek the best optimal cutoff using the R package ‘Survminer’. Kaplan–Meier curve and ROC analysis were performed to estimate the efficiency of the thirteen-gene signature in the internal training cohort (Figure 2A), internal testing cohort (Figure 2B) and external testing cohort (Figure 2C). Patients showed significantly poorer OS in the high-risk group compared with the low-risk group (*P*<0.001). The areas under the ROC curve (AUCs) for 1-, 3-, and 5-year respectively for internal training cohort, internal testing cohort and external testing cohort were 0.769, 0.746, 0.763; 0.757, 0.635, 0.601; 0.637, 0.666, 0.646.

### Correlation of risk-score model and clinical features

**Figure 3:**
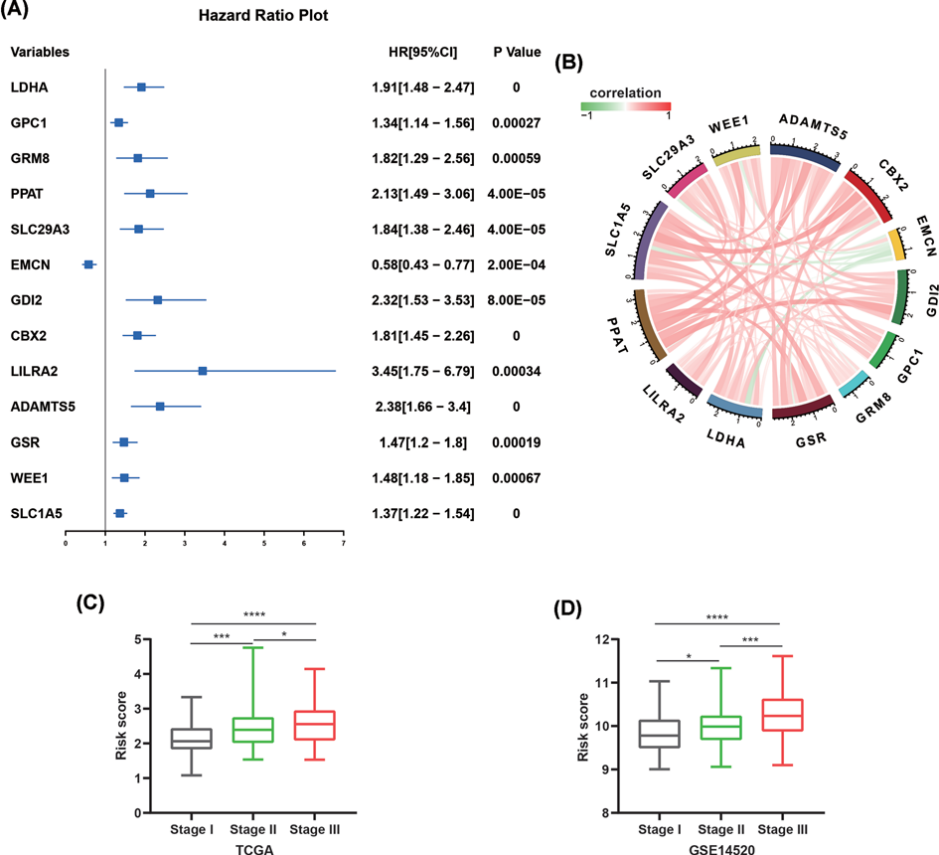
Correlation of risk-score model and WHO stages (**A**) The hazard ratios (HRs) with 95% confidence interval (95% CI) and *P*-value of univariate Cox regression were showed by Forest plot. (**B**) Correlation between thirteen genes, red and green curve respectively represents positive and negative correlation. (**C**) Risk score of samples in indicated stages in the TCGA cohort. (**D**) Risk score of samples in indicated stages in the GSE14520 cohort. Student’s t-test. *P <0.05, ***P<0.001, ****P<0.0001.

Forest map showed the univariate Cox regression analysis results of these thirteen genes (Figure 3A). The Kaplan–Meier survival analysis based on TCGA cohort revealed that EMCN was a favorable factor for prognosis (Supplementary Figure S1A), while the other 12 genes were risk factors for prognosis (Supplementary Figure S1B–M), which was consistent with the result of univariate Cox regression analysis. The mRNA expression level of each mRNA except EMCN, was higher in the high-risk group (Supplementary Figure S2A–M). Through correlation analysis, we found that the mRNA level of these 13 genes have a strong correlation: EMCN was negatively correlated with other 12 mRNAs, while these 12 mRNAs have positive correlation with each other (Figure 3B). In addition, we found that among the 13 genes in the prognosis model, 8 genes have been studied and proved to promote HCC progress, including CBX2 [9], SLC1A5 [10], LDHA [11], GSR [12], GPC1 [13], GDI2 [14], ADAMTS5 [15] and WEE1 [16]. While the other five genes have not been experimentally studied in HCC, including EMCN, LILRA2, PPAT, SLC29A3 and GRM8. We further verified the expression of these five genes using qRT-PCR. Our results showed that SLC29A3 and PPAT were high-expressed and EMCN was low-expressed in HCC tissues compared with adjacent normal tissues (Supplementary Figure S3A–C). There was no difference in LILRA2 and GRM8 expression between HCC tissues and adjacent normal tissues (Supplementary Figure S3D–W).

Moreover, we found that the risk score was higher in the high stage of HCC both in TCGA and GSE14520 cohort (Figure 3C,D). Univariate and multivariate Cox regression analyses were performed to evaluate the independent predictive ability of the risk-score model in TCGA HCC cohort. Results suggested the risk-score status, person neoplasm cancer status and tumor stage to act as independent prognostic factors (Table 1).

**Table 1:** The results of univariate and multivariate analyses

	Univariate analysis		Multivariate analysis	
TCGA LIHC cohort (*n*=366)	HR (95% CI)	*P*-value	HR (95% CI)	*P*-value
Neoplasm_histologic_grade (G3-4/G1-2)	1.083 (0.839–1.397)	0.542		
Person_neoplasm_cancer_status (With tumor/Tumor free)	1.877 (1.42–2.48)	<0.001	1.461 (1.081–1.975)	0.014
Relative_family_cancer_history (Yes/No)	1.125 (0.867–1.458)	0.375		
Vascular_tumor_cell_type (None/Micro or Macro)	0.808 (0.602–1.084)	0.155		
Gender (Male/Female)	0.867 (0.675–1.113)	0.263		
Age (>median/<median)	1.13 (0.881–1.448)	0.337		
Tumor_stage (Stage III+IV/I+II)	1.883 (1.448–2.448)	<0.001	1.423 (1.056–1.918)	0.02
Risk score (Low/High)	0.476 (0.364–0.614)	<0.001	0.503 (0.369–0.686)	<0.001
BCLC (B+C/A)	1.403 (0.851–2.311)	0.184		

### Construction and validation of predictive nomogram

**Figure 4:**
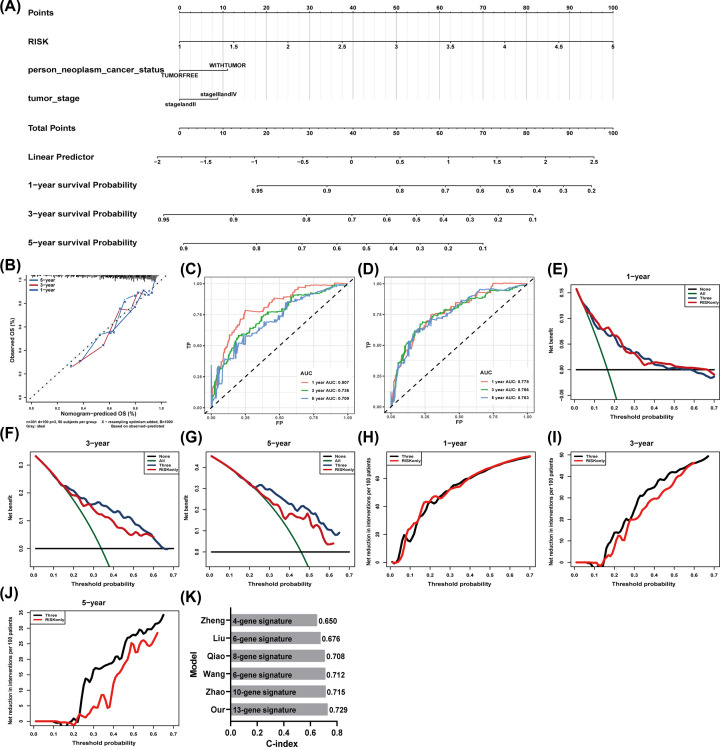
Construction of nomogram to predict OS of HCC patients (**A**) Nomogram for predicting probabilities of HCC patients with 1-, 3- and 5-year OS of HCC patients. (**B**) Calibration plot for predicting 1-, 3- and 5-year OS of HCC patient. Nomogram-based probability of survival is plotted on the x-axis; actual survival is plotted on the y-axis. (**C,D**) The ROC curve of the risk-score model (C) and nomogram model (D) for 1-, 3- and 5-year OS of HCC patient. (**E**–**J**) Net benefit (E–G) and net reduction (H–J) (y-axis) as calculated are plotted against the threshold probabilities of patients having 1-, 3- and 5-year survival on the x-axis. The green line represents the assumption that all patients have indicated survival time. The black solid line represents the assumption that no patients have indicated survival time. (**K**) The C-index values of prognostic models in indicated studies.

We further built a nomogram to predict OS according to the results of multivariate cox regression in TCGA HCC cohort (Figure 4A). The calibration curves for the 1-, 3- and 5-year OS showed good agreement between the prediction from the nomogram and the actual observations (Figure 4B). The AUCs for 1-, 3- and 5-year respectively for risk-score model and nomogram model in TCGA cohort were 0.807, 0.736, 0.709 (Figure 4C) and 0.778, 0.766, 0.763 (Figure 4D). The clinical usefulness was assessed using DCA. The results showed the nomogram model to perform better to help identify high-risk patients for intervention and low-risk patients to avoid over treatment, especially for 3- and 5-year prediction (Figure 4E–J). In addition, the C-index of our nomogram model was 0.729, which was higher than that in other five published models (Figure 4K) [17].

### Functional enrichment analysis

**Figure 5:**
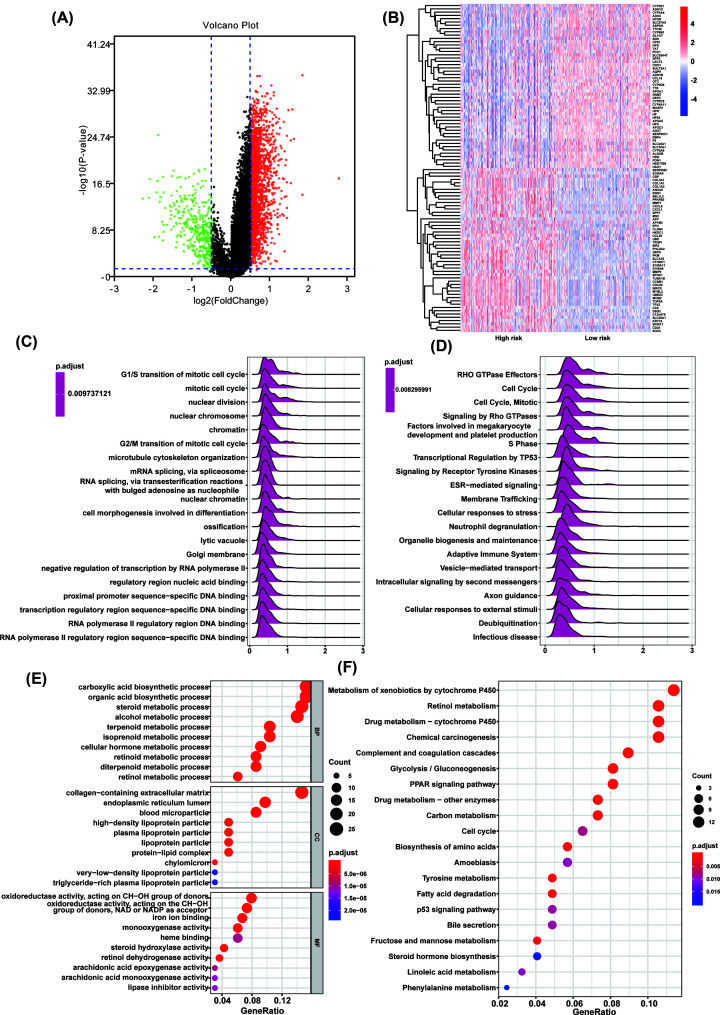
Function and pathway enrichment analysis of DEGs (**A**) The volcano plot of DEGs between high- and low-risk groups. (**B**) The heatmap showed the top 50 overexpressed genes in high-risk group and top 50 overexpressed genes in low-risk group. (**C,D**) Significant GSEA results of DEGs, including GO (C) and Reactome Pathways (D). (**E**) Significant GO terms of DEGs, including MF, CC and BP. (**F**) Significant KEGG pathways of DEGs.

To explore the differences between high- and low-risk groups, we divided the samples of TCGA HCC cohort into high- and low-risk groups according to the median of risk score. The volcano plot and heatmap showed the DEGs between high- and low-risk groups (Figure 5A,B). GSEA was conducted to search GO (Figure 5C) and Reactome Pathways (Figure 5D). Cell cycle-related terms were significantly enriched in high-risk group, such as G_1_/S transition of mitotic cell cycle, cell cycle and S phase terms. To further explore the potential functional pathways, functional pathway analysis was conducted based on the DEGs using R packages ‘clusterProfiler’. Results revealed that these genes were mainly associated with carboxylic acid biosynthetic process, organic acid biosynthetic process and steroid metabolic process in biological process (BP) category. For cellular component (CC) category, the DEGs were mainly enriched in collagen-containing extracellular matrix and endoplasmic reticulum lumen. In molecular function (MF) category, the DEGs were mainly enriched in oxidoreductase activity, acting on CH−OH group of donors (Figure 5E). Moreover, KEGG pathway analysis suggested the DEGs were enriched in various cross-talks in malignancy, including Metabolism of xenobiotics by cytochrome P450, Retinol metabolism, Drug metabolism–cytochrome P450, Glycolysis/Gluconeogenesis and PPAR signaling pathway (Figure 5F).

### TME analysis

**Figure 6:**
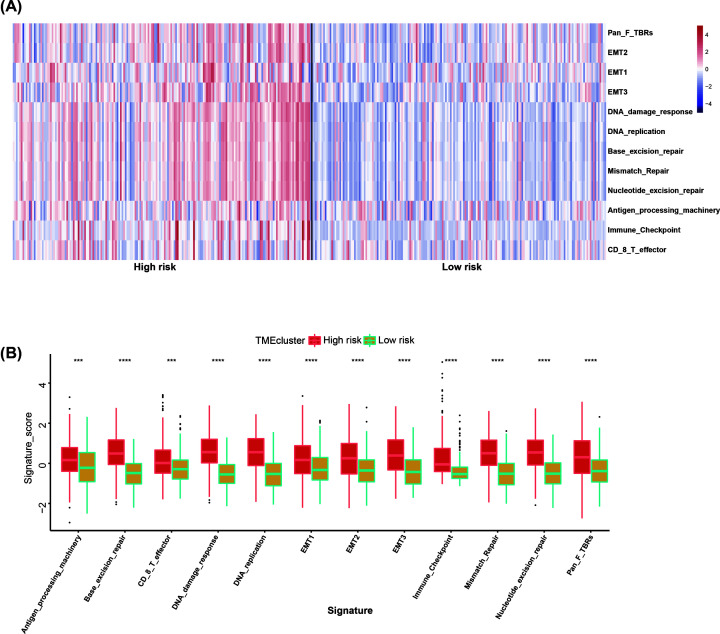
TME analysis of high- and low-risk groups (**A**) Heatmap of normalized (Z-score) score of TME signature. (**B**) The TME signature score of samples in high- and low-risk groups. Student’s t-test. ***P<0.001, ****P<0.0001.

**Figure 7:**
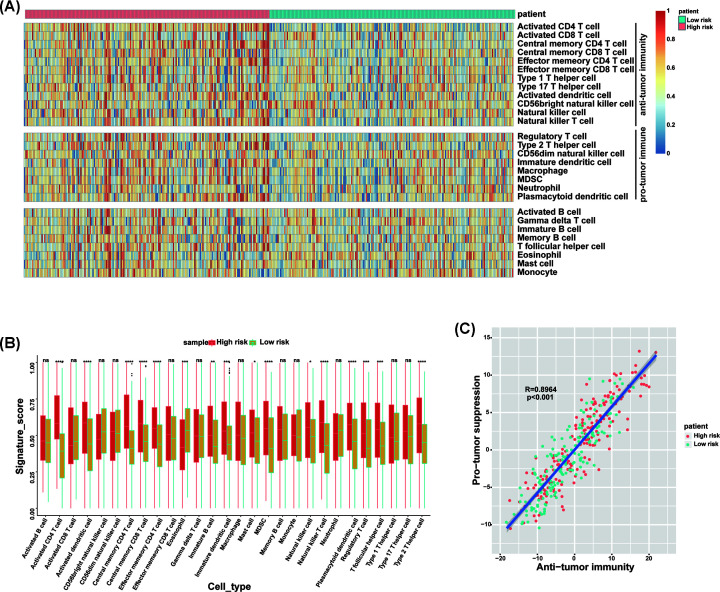
ssGSEA of high- and low-risk groups (**A**) Heatmap of normalized (Z-score) score of 28 infiltrating immune cell populations. (**B**) The signature score of 28 infiltrating immune cells in high- and low-risk groups. (**C**) Correlation between infiltration of cell types executing anti-tumor immunity and cell types executing pro-tumor immune suppressive functions. R coefficient of Pearson’s correlation. The shaded area represents 95% confidence interval. Red points represent high-risk patients, green points represent low-risk patients.*P<0.05, **P<0.01, ***P<0.001, ****P<0.0001.

TME, especially, immune microenvironment plays a key role in the survival of tumor patients. We hypothesized that high-risk patients have more complex TME. To our surprise, our analyses revealed that high-risk group was significantly associated with all indicated TME signatures (Figure 6A,B). To evaluate the infiltration level of immune cell, we employed ssGSEA to get the relative infiltration score of 28 immune cells (Figure 7A). We observed two major features. (1) The high-risk group had more complex immune cell components than the low-risk group, including cells executing anti-tumor reactivity (activated CD4 T cells, activated dendritic cells, central memory CD4 T cells, central memory CD8 T cells, Natural killer cells and Natural killer T cells) and cells delivering pro-tumor suppression (immature dendritic cells, MDSCs, plasmacytoid dendritic cell, regulatory T cells and Type 2 T helper cell) (Figure 7B). (2) Pearson’s correlation analysis revealed the infiltration level of cells executing anti-tumor reactivity and delivering pro-tumor suppression was positively correlated within a local environment (Figure 7C). This observation suggests a presence of a feedback mechanism such that the recruitment or differentiation of cells specialized for immune suppression may be facilitated by anti-tumor inflammation.

## Discussion

HCC is one of the most malignant tumor types worldwide with its poor prognosis and lack of effective early detection methods [8,18,19]. Therefore, it is urgent to identify new prognostic markers and establish more accurate prognostic models. Compared with a single biomarker, the prognostic gene signatures and conventional clinical parameters may have better prediction effect. In recent years, gene markers based on mRNAs have been widely used in tumor prognosis prediction [20–23]. However, prognostic gene signatures that could be used to show therapeutic effects is still very few.

In our study, we built a thirteen-gene prognostic model that included LDHA, GPC1, GRM8, PPAT, SLC29A3, EMCN, GDI2, CBX2, LILRA2, ADAMTS5, GSR, WEE1 and SLC1A5. The prediction performance of this thirteen-gene prognostic model was eligible not only in internal training set and internal testing set from TCGA HCC cohort, but also in external testing set from GSE14520 HCC cohort. The thirteen-gene risk model was also proved to be an independent prognostic factor for HCC. Patients in high-risk group have significantly worse OS than patients in low-risk group.

Nomogram is a tool to provide individual patients with the OS probability [24,25]. In our study, we built a nomogram model using the thirteen-gene signature combined with clinical prognostic factors. The calibration plots showed the actual survival rate to be in good agreement with the predicted survival rate, illustrating that the prediction performance of nomogram is eligible. We further proved the nomogram to be better than a single risk score factor by DCA plot. In addition, the C-index of our nomogram model was 0.729, which was higher than that in other five published models.

Since our thirteen-gene signature could effectively predict the OS in HCC patients, we further explore the differences between high- and low-risk samples. We divided the samples of TCGA HCC cohort into high- and low-risk groups according to the median of risk score. GSEA results showed that cell cycle-related pathways were significantly enriched in high-risk group. Cell cycle block or misoperation play an important role in the development of HCC [26–29]. Our results suggested the poor prognosis of high-risk group to be mainly due to the high level of cell cycle block or misoperation.

TME cells are an important part of tumor tissue. Increasing evidence has demonstrated their clinicopathological significance in predicting prognosis and efficacy [30,31]. TME signature based on characteristic gene expression is a new tool for evaluating comprehensive TME and a powerful biomarker for predicting survival and guiding more effective immunotherapy strategies [32]. Our results proved that high-risk group was significantly associated with many TME signatures, especially immune relevant signatures. By investigating the regulation of immune cell infiltration level, we observed two major features. (1) The high-risk group had more complex immune cell components than the low-risk group, including cells executing anti-tumor reactivity (activated CD4 T cells, activated dendritic cells, central memory CD4 T cells, central memory CD8 T cells, Natural killer cells and Natural killer T cells) and cells delivering pro-tumor suppression (immature dendritic cells, MDSCs, plasmacytoid dendritic cell, regulatory T cells and Type 2 T helper cell). (2) Pearson’s correlation analysis revealed the infiltration level of these two categories of cells to be positively correlated within a local environment. Based on the results of TME analysis, we believed that it is not enough to use only one or a class of TME components to predict the prognosis, but also depends on the overall situation of TME. The more complex the TME, the higher the risk of tumor and the worse the prognosis. Our conclusion is consistent with that of the previously reported article [33].

Overall, our research indicated that the thirteen-gene signature prognostic model is a trustworthy tool for the prediction of OS in HCC. Further construction of nomogram with this thirteen-gene signature prognostic model could assist clinicians to choose personalized treatment for patients with HCC.

## Supplementary Material

Supplementary Figures S1-S3 and Tables S1-S3

## Data Availability

All data, models, or code used in the present study are available from the corresponding authors on request.

## Abbreviations

AUC: area under the ROC curve
DCA: decision curve analysis
DEG: differentially expressed gene
GEO: Gene Expression Omnibus
GO: Gene Ontology
GSEA: Gene Set Enrichment Analysis
GSVA: Gene Set Variation Analysis
HCC: hepatocellular carcinoma
KEGG: Kyoto Encyclopedia of Genes and Genomes
OS: overall survival
ROC: receiver operating characteristic
ssGSEA: single-sample GSEA
TCGA: The Cancer Genome Atlas
TME: tumor microenvironment
