# Supplementary material for: Identification of a thirteen-gene signature predicting overall survival for hepatocellular carcinoma
Source: Biosci Rep. 2021 Apr 22;41(4):BSR20202870. doi: 10.1042/BSR20202870 (PMC8065179; doi:10.1042/BSR20202870)
Supplement: Supplementary Figures S1-S3 and Tables S1-S3 [file BSR-2020-2870_supp.pdf]

Supplementary figures and figure legends

Sup-Figure 1: Overall survival analysis of 13 genes.

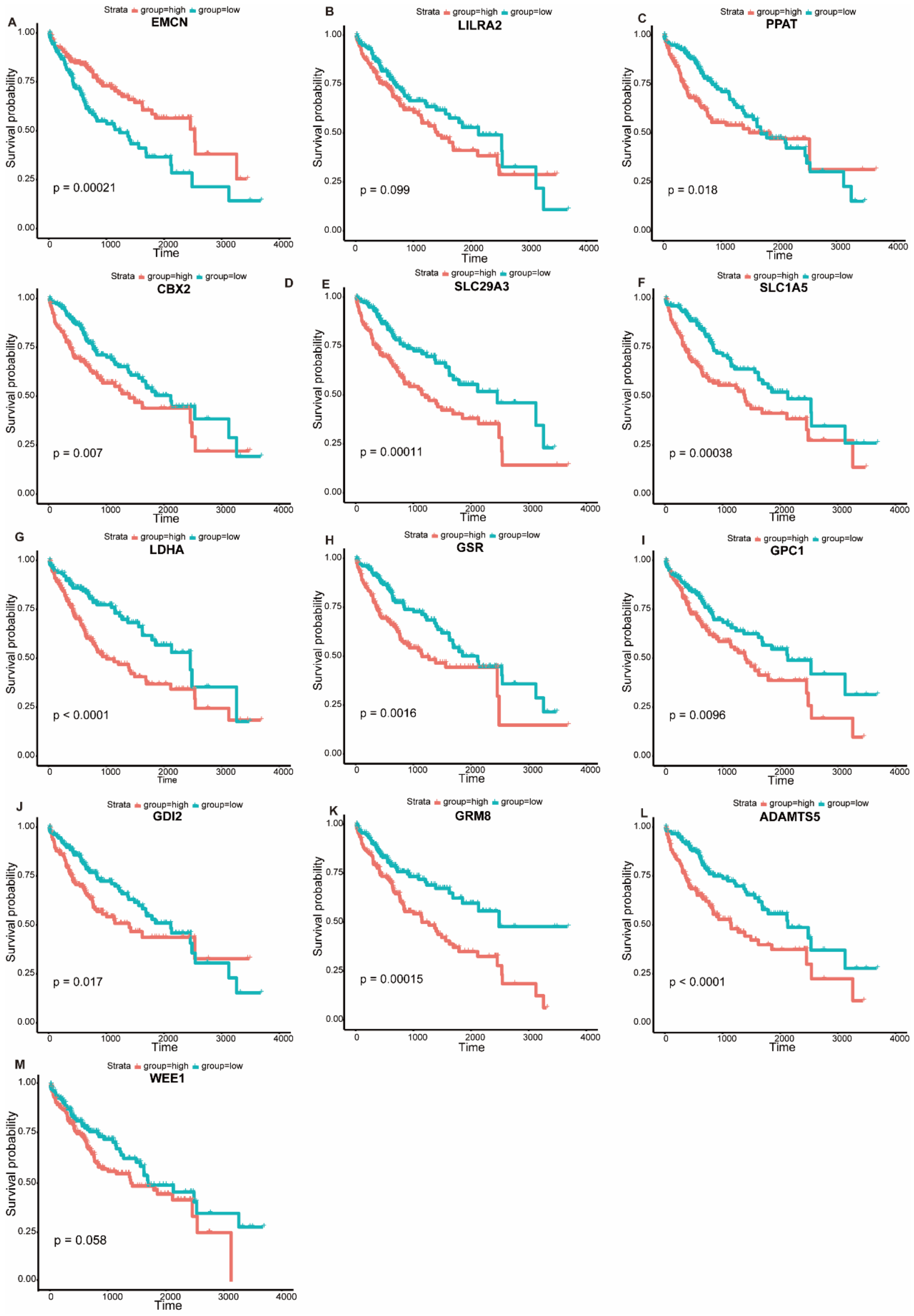

Sup-Figure 1: A-M, Kaplan-Meier analysis of OS in line with the expression of thirteen genes in TCGA cohort.

Sup-Figure 2: The expression of 13 genes in TCGA cohort.

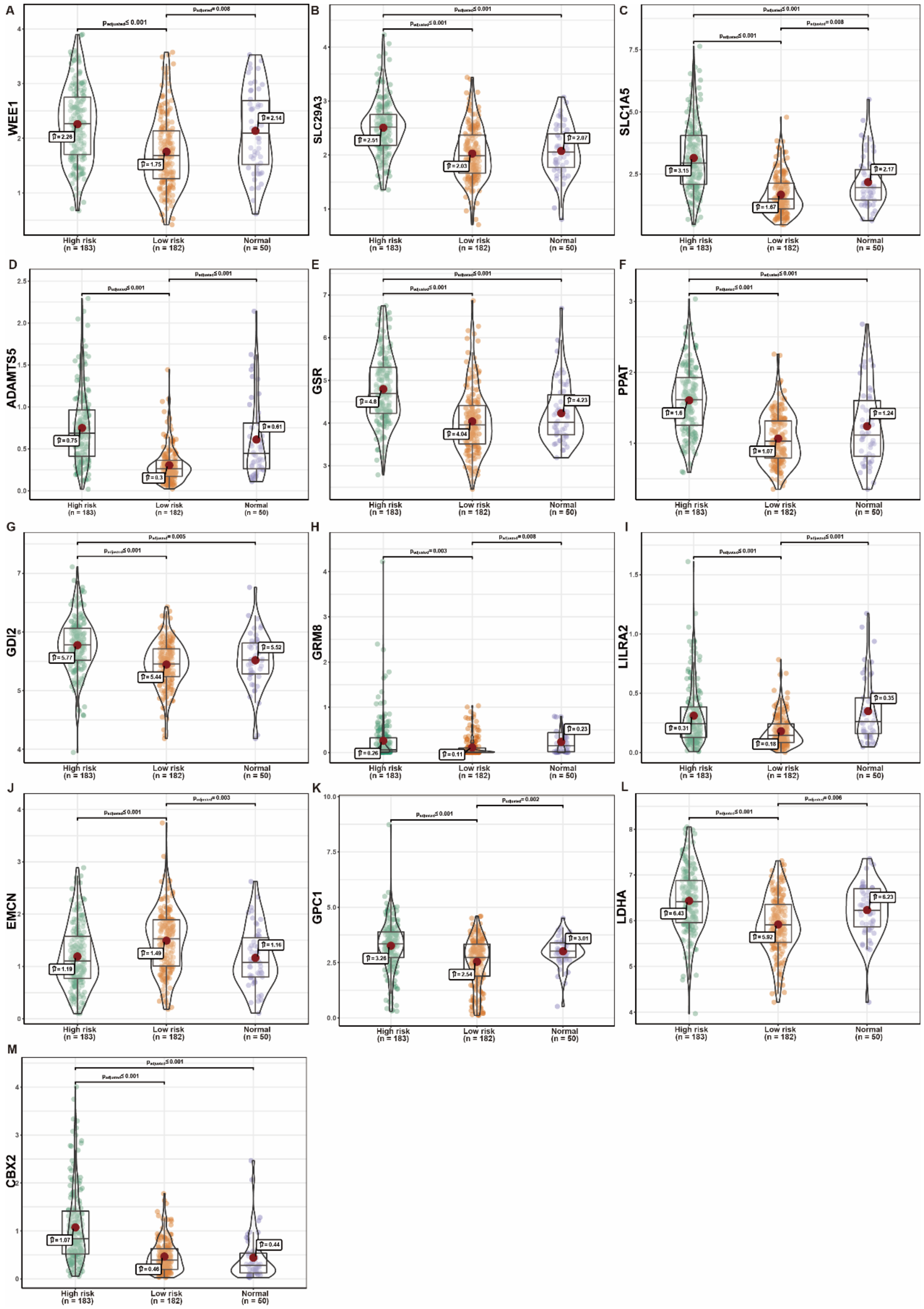

Sup-Figure 2: A-M, The expression value of thirteen genes in high-risk, low-risk, and normal sample groups in TCGA cohort.

Sup-Figure 3: The expression of 5 genes in HCC patients.

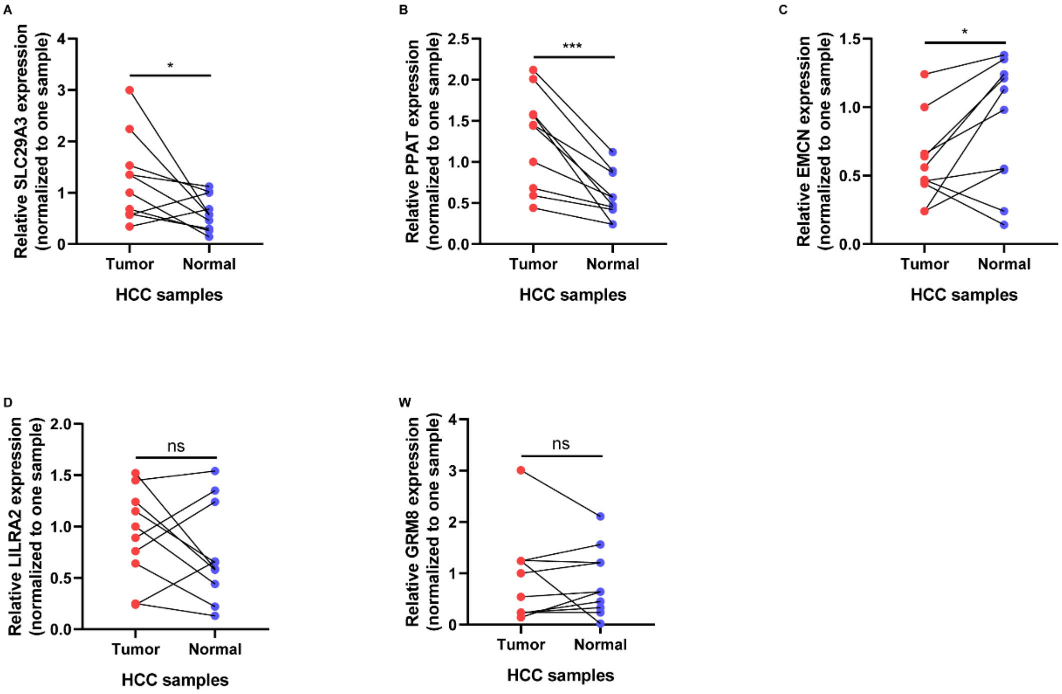

Sup-Figure 3: A-W, The relative mRNA expression of indicated genes in HCC tumor and adjacent normal tissues.

Supplementary Table 1: Baseline data

	GSE14520 (221 with RNA seq)	TCGA (371 with RNA seq)
**Overall status**		
**Dead**	85	130
**Alive**	136	241
**WHO stages**		
**Stage I**	93	171
**Stage II**	77	86
**Stage III**	49	85
**Stage IV**	0	5
**Unkown**	2	24
**Age**		
**>60**	43	201
**<60**	178	169
**Unknow**	0	1
**Gender**		
**Male**	191	250
**Female**	30	121

Supplementary Table 1: Clinical baseline data of patients from GSE14520 and TCGA

Supplementary Table 2: Primer sequences of 5 genes

Gene names	Primer sequences
ACTB Forward Primer	CATGTACGTTGCTATCCAGGC
ACTB Reverse Primer	CTCCTTAATGTCACGCACGAT
EMCN Forward Primer	AGCAACCAGCCGGTCTTATTC
EMCN Reverse Primer	AGCACATTCGGTACAAACCCA
LILRA2 Forward Primer	AGCCCCAGGAAAGAACGTG
LILRA2 Reverse Primer	GCTGAGTGAGCTGTAGCATCT
PPAT Forward Primer	GATGGGAGTTCGGTGCCAA
PPAT Reverse Primer	CAACGAAGGGCTGACAATTTTC
SLC29A3 Forward Primer	TTGAGAGCTACCTTGCCGTTG
SLC29A3 Reverse Primer	CAGTGCAGTTATCACCATGAAGA
GRM8 Forward Primer	CGAGGGAAAGCGATCAGCC
GRM8 Reverse Primer	CCCATCCACCCGTATGGAA

Supplementary Table 2: The primer sequences of indicated genes.

Supplementary Table 3: Univariate cox results

gene name	Hazard_Ratio	CI95	P_Value
LDHA	1.91	1.48 - 2.47	0
GPC1	1.34	1.14 - 1.56	0.00027
GRM8	1.82	1.29 - 2.56	0.00059
PPAT	2.13	1.49 - 3.06	4.00E-05
SLC29A3	1.84	1.38 - 2.46	4.00E-05
EMCN	0.58	0.43 - 0.77	2.00E-04
GDI2	2.32	1.53 - 3.53	8.00E-05
CBX2	1.81	1.45 - 2.26	0
LILRA2	3.45	1.75 - 6.79	0.00034
ADAMTS5	2.38	1.66 - 3.4	0
GSR	1.47	1.2 - 1.8	0.00019
WEE1	1.48	1.18 - 1.85	0.00067
SLC1A5	1.37	1.22 - 1.54	0
AAMP	1.91	1.3 - 2.8	0.00092
ABCC5	1.93	1.35 - 2.75	0.00028
ACYP1	1.87	1.34 - 2.59	2.00E-04
ADM	1.31	1.13 - 1.52	0.00044
ADSL	1.65	1.23 - 2.22	9.00E-04
AMD1	2.02	1.51 - 2.71	0
ANP32B	1.62	1.24 - 2.12	0.00042
ANXA5	1.36	1.15 - 1.6	0.00032
ARL8B	1.81	1.28 - 2.57	0.00085
ARMC9	2.19	1.39 - 3.44	0.00067
ASF1A	1.64	1.22 - 2.19	0.00095
ATIC	1.55	1.2 - 2.01	8.00E-04
ATP13A2	1.56	1.23 - 1.97	0.00024
ATP1B3	1.33	1.14 - 1.56	4.00E-04
ATP6V0A4	93.65	6.36 - 1379.86	0.00094
B4GALT5	1.48	1.22 - 1.81	9.00E-05
BARD1	1.89	1.34 - 2.66	0.00028
BBS7	2.34	1.54 - 3.55	6.00E-05
BCAT1	1.52	1.21 - 1.92	0.00038
BCL10	1.8	1.31 - 2.46	0.00026
BCORL1	1.62	1.24 - 2.13	0.00047
BDH1	0.76	0.66 - 0.88	0.00014
BFSP1	2.93	1.76 - 4.86	3.00E-05
BICD1	2.73	1.61 - 4.63	2.00E-04
BIRC5	1.25	1.1 - 1.42	0.00063
BRPF1	2.02	1.36 - 2.99	0.00045
BUB1	1.57	1.26 - 1.96	6.00E-05

BUB1B	1.49	1.22 - 1.83	0.00011
C11orf16	3296.14	77.63 - 139961.77	2.00E-05
C19orf26	12.35	5.5 - 27.71	0
C2orf43	2.26	1.51 - 3.38	7.00E-05
C2orf44	2.07	1.36 - 3.13	0.00065
C3orf52	2.19	1.46 - 3.31	0.00018
C5orf30	1.56	1.27 - 1.91	3.00E-05
CACNA1B	4.74	2.09 - 10.78	2.00E-04
CALCR	2.12	1.38 - 3.28	0.00068
CAP1	1.78	1.31 - 2.43	0.00026
CARD9	2.44	1.47 - 4.03	0.00052
CASP2	1.64	1.22 - 2.19	0.00092
CCNA2	1.27	1.11 - 1.46	0.00053
CCNB1	1.29	1.12 - 1.49	0.00039
CCNF	1.63	1.26 - 2.11	0.00022
CCNJL	2.38	1.75 - 3.22	0
CCT2	1.73	1.3 - 2.31	0.00016
CCT4	1.78	1.31 - 2.43	0.00026
CCT5	1.72	1.34 - 2.2	2.00E-05
CCT7	1.71	1.24 - 2.36	0.00099
CD3EAP	1.76	1.27 - 2.45	0.00077
CD68	1.55	1.2 - 2	0.00078
CD97	1.35	1.15 - 1.59	0.00025
CDC20	1.27	1.13 - 1.43	5.00E-05
CDC25A	1.58	1.24 - 2.01	0.00025
CDC25B	1.38	1.17 - 1.64	0.00014
CDC42EP2	1.51	1.2 - 1.9	4.00E-04
CDC7	1.61	1.26 - 2.07	0.00016
CDCA3	1.53	1.23 - 1.92	0.00018
CDCA4	1.55	1.23 - 1.96	0.00024
CDCA8	1.49	1.26 - 1.76	0
CDH10	1.69	1.24 - 2.3	8.00E-04
CDK4	1.44	1.18 - 1.77	0.00038
CDR1	526.59	16.2 - 17121.84	0.00042
CDV3	1.84	1.32 - 2.55	3.00E-04
CENPA	1.59	1.31 - 1.92	0
CENPE	2.09	1.53 - 2.86	0
CENPI	2.01	1.44 - 2.82	4.00E-05
CENPO	1.78	1.33 - 2.37	9.00E-05
CEP55	1.64	1.36 - 1.99	0
CFHR4	0.8	0.72 - 0.89	5.00E-05
CHEK1	1.79	1.35 - 2.38	5.00E-05
CHODL	1.54	1.24 - 1.91	1.00E-04

CHORDC1	2.26	1.58 - 3.24	1.00E-05
CIT	2.16	1.51 - 3.08	2.00E-05
CKAP5	1.68	1.27 - 2.23	0.00029
CLCF1	1.41	1.15 - 1.71	0.00073
CLCN2	1.69	1.27 - 2.25	0.00037
CLDN6	1.71	1.29 - 2.28	0.00022
CLEC3B	0.73	0.62 - 0.86	0.00012
CLSPN	2.17	1.52 - 3.09	2.00E-05
CNOT6	1.8	1.33 - 2.44	0.00016
CORO1C	1.59	1.22 - 2.07	0.00054
CPB2	0.85	0.77 - 0.94	0.00086
CPSF6	1.75	1.27 - 2.4	0.00061
CRH	2.06	1.39 - 3.06	3.00E-04
CRISP2	1.99	1.36 - 2.92	0.00043
CSF1	1.39	1.16 - 1.66	0.00027
CSF3R	1.52	1.21 - 1.9	0.00026
CSH1	1.09E+25	12076250795509.6 - 9.88344118950835e+36	4.00E-05
CSN1S1	391.95	34.32 - 4476.56	0
CSTF2	1.63	1.26 - 2.11	0.00017
CYP26B1	1.55	1.29 - 1.88	0
CYP2C9	0.88	0.82 - 0.95	0.00051
DAB2	1.4	1.19 - 1.64	3.00E-05
DBF4	2.12	1.51 - 2.99	2.00E-05
DDN	8.81	2.91 - 26.7	0.00012
DFNA5	1.65	1.28 - 2.14	0.00014
DNAJB4	1.49	1.18 - 1.89	0.00081
DPH2	1.61	1.22 - 2.13	0.00069
DR1	1.81	1.32 - 2.49	0.00024
DSG2	1.26	1.1 - 1.43	6.00E-04
DYNC1H1	1.64	1.24 - 2.19	0.00065
DYNC1LI1	2.6	1.79 - 3.77	0
E2F2	1.94	1.36 - 2.76	0.00024
E2F5	1.81	1.34 - 2.45	0.00011
E2F6	2.27	1.53 - 3.36	5.00E-05
ECM2	0.73	0.61 - 0.88	0.00077
ECT2	1.41	1.18 - 1.68	0.00017
EGLN3	1.32	1.13 - 1.55	0.00036
EIF1B	1.98	1.35 - 2.92	0.00052
EIF2B5	2.2	1.45 - 3.35	0.00022
EIF2S1	2.1	1.37 - 3.21	0.00062
EIF5B	1.86	1.35 - 2.57	0.00015

EMR3	3.01	1.67 - 5.43	0.00026
ENO1	1.52	1.27 - 1.82	0
EPO	1.29	1.16 - 1.44	1.00E-05
ESPL1	1.51	1.18 - 1.92	0.00087
ETF1	2.12	1.43 - 3.14	0.00018
ETV5	1.58	1.28 - 1.95	2.00E-05
EXO1	1.57	1.24 - 2	0.00021
EZH2	1.58	1.27 - 1.96	5.00E-05
FABP6	1.46	1.2 - 1.78	0.00019
FAM90A1	2.72	1.54 - 4.81	6.00E-04
FANCE	1.6	1.25 - 2.06	0.00025
FANCL	2.02	1.43 - 2.85	7.00E-05
FBXO5	2.03	1.52 - 2.7	0
FGF9	11.2	3.55 - 35.38	4.00E-05
FHOD3	1.67	1.29 - 2.15	8.00E-05
FOXI1	6.47	2.5 - 16.78	0.00012
FOXJ3	1.85	1.32 - 2.6	0.00033
FOXK2	1.81	1.32 - 2.49	0.00024
FOXM1	1.31	1.12 - 1.52	0.00054
FTCD	0.86	0.79 - 0.94	4.00E-04
FUBP1	1.6	1.23 - 2.07	0.00037
FZD3	4.01	1.85 - 8.69	0.00043
FZD7	1.5	1.24 - 1.82	4.00E-05
G6PD	1.33	1.19 - 1.5	0
GAD1	2.23	1.42 - 3.51	0.00049
GALNS	1.67	1.26 - 2.21	0.00038
GINS1	1.47	1.22 - 1.76	5.00E-05
GLMN	2.17	1.46 - 3.22	0.00013
GLP1R	1.7	1.26 - 2.29	0.00055
GMEB1	2.22	1.4 - 3.53	0.00069
GMPS	1.73	1.28 - 2.33	0.00034
GNAI3	2.11	1.38 - 3.25	0.00064
GNG5	1.71	1.28 - 2.29	0.00032
GNL2	2.05	1.45 - 2.89	5.00E-05
GNPDA1	1.56	1.25 - 1.95	1.00E-04
GOT2	0.68	0.55 - 0.84	0.00049
GPD2	1.77	1.32 - 2.37	0.00015
GPRC5D	3.54	2.07 - 6.08	0
GPSM2	2.06	1.49 - 2.86	2.00E-05
GRID2	15047.13	173.48 - 1305138.65	2.00E-05
GRM4	18.63	4.23 - 82.01	0.00011
GTDC1	3.33	1.85 - 5.99	6.00E-05
GTF2H1	2.16	1.45 - 3.23	0.00017

GTF3C2	1.77	1.29 - 2.44	0.00044
GTPBP4	1.89	1.42 - 2.51	1.00E-05
GTSE1	1.6	1.29 - 1.97	1.00E-05
H2AFY	1.7	1.26 - 2.3	6.00E-04
HAND1	17.05	3.91 - 74.31	0.00016
HAT1	1.82	1.28 - 2.6	0.00085
HAVCR1	1.5	1.28 - 1.75	0
HDAC1	1.67	1.27 - 2.2	0.00024
HDAC2	2.1	1.54 - 2.87	0
HECTD3	1.73	1.25 - 2.37	0.00079
HMMR	1.47	1.22 - 1.77	5.00E-05
HN1	1.34	1.14 - 1.57	0.00036
HOXC5	104.46	11.41 - 956.53	4.00E-05
HOXD10	1.95	1.38 - 2.76	0.00015
HOXD13	2.26	1.5 - 3.4	1.00E-04
HRASLS	2.47	1.46 - 4.17	0.00077
HSF2	1.7	1.26 - 2.3	0.00056
HSPA14	1.88	1.32 - 2.69	0.00047
HTR5A	32.24	6.31 - 164.64	3.00E-05
HTRA2	2.1	1.4 - 3.15	0.00036
ICMT	1.67	1.24 - 2.25	0.00084
IGFBP3	1.23	1.09 - 1.39	0.00082
IGSF3	1.35	1.15 - 1.57	0.00018
IL12A	4.08	1.93 - 8.61	0.00023
IL15RA	1.55	1.28 - 1.89	1.00E-05
IMPDH1	1.4	1.2 - 1.64	2.00E-05
IQCC	1.99	1.36 - 2.92	0.00044
KATNA1	1.91	1.34 - 2.74	0.00041
KIF11	1.47	1.2 - 1.81	0.00022
KIF14	1.66	1.23 - 2.23	0.00092
KIF18A	2.07	1.56 - 2.75	0
KIF20A	1.52	1.28 - 1.82	0
KIF23	1.59	1.27 - 1.98	5.00E-05
KIF2A	1.69	1.24 - 2.28	0.00076
KIF2C	1.46	1.25 - 1.72	0
KIF4A	1.36	1.16 - 1.61	0.00022
KIFC1	1.29	1.12 - 1.5	0.00055
KLHL18	2.7	1.53 - 4.77	0.00062
KRT17	1.29	1.12 - 1.49	0.00032
KRT32	14892196	1550.71 - 143016857397.09	0.00042
KRT34	5.49	2.17 - 13.87	0.00031
KRT38	159370.5	404.22 - 62834351.15	9.00E-05

LAPTM4B	1.25	1.09 - 1.42	0.00096
LHFPL2	1.53	1.21 - 1.92	0.00031
LIMS2	0.62	0.48 - 0.79	0.00014
LMNB1	1.36	1.16 - 1.59	0.00019
LMNB2	1.45	1.19 - 1.78	0.00026
LMO4	1.6	1.24 - 2.07	0.00029
LRCH3	2.21	1.39 - 3.53	0.00085
LRP10	1.5	1.19 - 1.89	0.00052
LRP12	1.85	1.37 - 2.51	6.00E-05
LRRC41	2.04	1.43 - 2.92	9.00E-05
MAD2L1	1.53	1.21 - 1.94	0.00042
MAPK7	1.87	1.33 - 2.64	0.00036
MAPKAPK5	2.41	1.45 - 4.01	0.00072
MARCKSL1	1.28	1.12 - 1.47	0.00039
MCM10	1.97	1.5 - 2.57	0
MCM2	1.31	1.13 - 1.52	0.00046
MCM6	1.37	1.15 - 1.64	5.00E-04
ME2	1.73	1.26 - 2.36	6.00E-04
MED8	2.22	1.56 - 3.16	1.00E-05
MELK	1.41	1.18 - 1.69	2.00E-04
MGMT	0.7	0.56 - 0.86	0.00086
MKI67	1.37	1.15 - 1.63	0.00032
MMP1	1.52	1.29 - 1.79	0
MPP2	3.47	1.97 - 6.13	2.00E-05
MRPL3	1.89	1.32 - 2.7	0.00055
MTBP	3.29	1.76 - 6.14	0.00018
MTCP1	2.04	1.42 - 2.95	0.00014
MTF2	1.78	1.3 - 2.43	0.00034
MTMR2	1.86	1.41 - 2.45	1.00E-05
MUTYH	1.84	1.34 - 2.53	0.00018
MYBL2	1.22	1.1 - 1.36	0.00021
MYH6	10.74	3.97 - 29.02	0
MYOD1	32.51	8.67 - 121.83	0
NAP1L1	1.43	1.16 - 1.77	0.00098
NAP1L4	1.87	1.32 - 2.66	0.00048
NASP	1.51	1.19 - 1.93	0.00079
NAT10	1.84	1.29 - 2.63	0.00085
NCAPD2	1.44	1.18 - 1.76	0.00038
NCAPG	1.51	1.24 - 1.83	3.00E-05
NCAPG2	1.56	1.22 - 1.99	0.00044
NCAPH	1.42	1.17 - 1.73	0.00037
NCBP2	1.73	1.27 - 2.35	0.00054
NCF2	1.32	1.12 - 1.54	0.00064

NCL	1.92	1.39 - 2.66	9.00E-05
NDRG1	1.26	1.1 - 1.44	0.00075
NDUFAF1	0.5	0.34 - 0.74	0.00054
NEIL3	1.98	1.51 - 2.58	0
NME7	1.94	1.32 - 2.84	0.00069
NOL10	2.04	1.4 - 2.98	0.00022
NPC1	1.58	1.21 - 2.06	0.00082
NR0B1	1.46	1.21 - 1.76	8.00E-05
NRAS	1.58	1.22 - 2.05	0.00061
NUP107	1.76	1.29 - 2.4	0.00034
NUP155	1.76	1.29 - 2.4	0.00039
NUP205	1.75	1.32 - 2.33	0.00011
NUP43	1.78	1.3 - 2.43	0.00032
NUP85	1.76	1.32 - 2.36	0.00014
OPRK1	2.72	1.67 - 4.43	6.00E-05
OR10J1	1.22E+19	239704824.17 - 6.25689795763118e+29	0.00048
OSBP2	1.63	1.22 - 2.18	0.00085
P2RY4	2.28	1.49 - 3.47	0.00013
P2RY6	1.64	1.28 - 2.1	8.00E-05
PAGE1	1.2	1.1 - 1.3	2.00E-05
PBK	1.37	1.16 - 1.62	0.00018
PCBP2	2.22	1.54 - 3.19	2.00E-05
PDE6A	611.5	36.74 - 10178.24	1.00E-05
PDK3	1.68	1.27 - 2.22	0.00025
PFKFB4	1.81	1.42 - 2.31	0
PFN2	1.35	1.19 - 1.53	0
PGLYRP4	13.47	3.6 - 50.43	0.00011
PHC2	1.69	1.28 - 2.25	0.00024
PHKA2	1.72	1.29 - 2.3	0.00023
PIAS2	2.76	1.52 - 5.02	0.00087
PLAUR	1.38	1.15 - 1.66	0.00063
PLK1	1.45	1.22 - 1.72	2.00E-05
PLOD2	1.51	1.25 - 1.83	2.00E-05
PNMA2	2.11	1.43 - 3.12	0.00017
POF1B	1.35	1.15 - 1.58	0.00018
POLA1	1.78	1.29 - 2.45	4.00E-04
POLQ	2.39	1.52 - 3.77	0.00017
POLR3G	2.52	1.57 - 4.04	0.00013
PON1	0.86	0.8 - 0.93	0.00011
POU3F2	4.58	1.94 - 10.78	5.00E-04
PPM1G	1.79	1.33 - 2.41	0.00013

PPP1CB	1.79	1.32 - 2.42	0.00018
PPT1	1.54	1.24 - 1.91	9.00E-05
PRDM14	16917824	28399.58 - 10078062417.21	0
PRDX1	1.53	1.2 - 1.96	0.00063
PRKCD	1.42	1.16 - 1.75	0.00067
PRKRA	1.99	1.37 - 2.89	0.00032
PRPF19	2.12	1.43 - 3.14	0.00019
PRR11	1.65	1.34 - 2.03	0
PSMB2	1.76	1.28 - 2.42	0.00055
PSMC3IP	1.92	1.4 - 2.63	6.00E-05
PSMD1	2.03	1.37 - 2.99	0.00038
PSMD14	1.76	1.28 - 2.44	6.00E-04
PSRC1	1.72	1.36 - 2.16	0
PTP4A2	1.92	1.4 - 2.64	5.00E-05
RAB32	1.68	1.34 - 2.1	1.00E-05
RAB3IL1	1.42	1.2 - 1.68	6.00E-05
RACGAP1	1.39	1.14 - 1.69	0.00089
RAD1	1.84	1.3 - 2.6	6.00E-04
RAD51AP1	1.42	1.16 - 1.76	0.00092
RAD54B	4.46	2.2 - 9.03	3.00E-05
RAD54L	1.57	1.25 - 1.99	0.00013
RAE1	1.79	1.28 - 2.52	0.00077
RAMP3	0.72	0.61 - 0.85	9.00E-05
RAN	1.66	1.29 - 2.13	8.00E-05
RANBP1	1.67	1.29 - 2.17	0.00012
RAX	291.4	11.5 - 7384.74	0.00058
RBBP4	1.61	1.23 - 2.1	0.00055
RBM14	2.05	1.36 - 3.07	0.00054
RBM17	1.94	1.43 - 2.64	2.00E-05
RBM28	2.72	1.61 - 4.6	2.00E-04
REEP4	1.43	1.16 - 1.76	0.00076
RGS17	3.23	1.62 - 6.43	0.00088
RGS2	1.21	1.08 - 1.35	0.00079
RIBC2	1.53	1.21 - 1.95	0.00052
RIMS3	2.53	1.57 - 4.09	0.00015
RIT1	1.66	1.26 - 2.19	3.00E-04
RNF186	2.26	1.53 - 3.32	4.00E-05
RNF2	1.96	1.44 - 2.68	2.00E-05
RORC	0.79	0.7 - 0.9	0.00052
RPS6KA6	3.11	1.59 - 6.09	0.00091
RQCD1	1.79	1.31 - 2.43	0.00024
RRAGC	1.81	1.35 - 2.43	6.00E-05

RTN3	1.88	1.4 - 2.51	3.00E-05
RUVBL1	1.95	1.42 - 2.68	3.00E-05
S100A8	1.24	1.09 - 1.4	0.00064
S100A9	1.25	1.15 - 1.36	0
S100PBP	1.99	1.33 - 2.96	0.00074
SAP30	1.66	1.3 - 2.14	7.00E-05
SCRN1	1.31	1.12 - 1.52	0.00049
SDC3	1.41	1.15 - 1.74	0.00099
SEMA6A	1.32	1.13 - 1.54	0.00045
SEPHS1	2.26	1.56 - 3.27	2.00E-05
SERBP1	1.98	1.36 - 2.89	4.00E-04
SF3A3	2.05	1.47 - 2.86	2.00E-05
SFPQ	1.88	1.4 - 2.53	3.00E-05
SGCB	1.35	1.13 - 1.62	0.00093
SHCBP1	1.56	1.23 - 1.97	0.00024
SIGLEC9	1.75	1.27 - 2.4	0.00062
SLC11A1	1.53	1.2 - 1.96	0.00067
SLC16A3	1.35	1.17 - 1.55	3.00E-05
SLC1A6	74.66	6.18 - 902.28	0.00069
SLC1A7	1.27	1.12 - 1.44	0.00024
SLC25A22	1.72	1.28 - 2.3	0.00031
SLC25A24	1.54	1.24 - 1.91	9.00E-05
SLC2A1	1.49	1.27 - 1.74	0
SLC2A2	0.87	0.8 - 0.94	8.00E-04
SLC36A1	1.86	1.35 - 2.55	0.00013
SLC38A1	1.28	1.12 - 1.48	0.00049
SLC7A8	1.53	1.25 - 1.86	3.00E-05
SMOX	1.37	1.16 - 1.62	0.00017
SMS	1.82	1.42 - 2.34	0
SNX5	1.96	1.37 - 2.82	0.00026
SNX7	1.45	1.21 - 1.74	5.00E-05
SOX11	7.34	2.4 - 22.48	0.00048
SPA17	2.08	1.41 - 3.07	0.00021
SPANXC	33.52	6.48 - 173.47	3.00E-05
SPINK4	1.37	1.14 - 1.66	0.00093
SPP1	1.11	1.05 - 1.17	0.00026
SSRP1	1.82	1.36 - 2.43	5.00E-05
STAM	1.72	1.25 - 2.36	0.00087
STC2	1.39	1.18 - 1.64	1.00E-04
STIP1	1.56	1.2 - 2.01	0.00074
STMN1	1.29	1.11 - 1.5	0.00075
STX3	1.48	1.21 - 1.82	0.00014
STXBP5L	2003.57	41.07 - 97732.78	0.00013

SUV39H2	1.94	1.37 - 2.73	0.00017
TACR3	214.82	17.16 - 2688.4	3.00E-05
TAF1B	2.09	1.42 - 3.07	0.00017
TARDBP	2.65	1.56 - 4.5	3.00E-04
TARS	1.8	1.34 - 2.42	1.00E-04
TBL1XR1	1.73	1.27 - 2.38	0.00062
TBX19	2.16	1.39 - 3.38	0.00068
TCOF1	1.73	1.3 - 2.29	0.00013
TEX15	7.02	3.39 - 14.57	0
TFDP1	1.45	1.18 - 1.78	0.00044
TIMM23	2.11	1.41 - 3.16	0.00029
TMEM39B	2.17	1.45 - 3.24	0.00016
TMPRSS6	0.84	0.76 - 0.93	0.00055
TNFRSF11A	1.92	1.37 - 2.69	0.00013
TNFRSF11B	1.26	1.1 - 1.44	0.00068
TOE1	2.15	1.48 - 3.13	6.00E-05
TPX2	1.36	1.18 - 1.57	2.00E-05
TRAPPC4	1.66	1.24 - 2.23	0.00059
TREM1	1.59	1.27 - 1.99	4.00E-05
TRIM36	3.12	1.8 - 5.4	5.00E-05
TRIP13	1.59	1.32 - 1.91	0
TROAP	1.33	1.12 - 1.57	0.00099
TRPC4AP	1.96	1.33 - 2.89	7.00E-04
TTC26	3.16	1.99 - 5.02	0
TTK	1.74	1.39 - 2.17	0
TUBG1	1.51	1.21 - 1.87	0.00022
TXLNA	1.79	1.3 - 2.47	0.00036
UAP1L1	1.4	1.19 - 1.64	3.00E-05
UBAP2	1.78	1.27 - 2.48	0.00075
UBE2E1	1.87	1.38 - 2.54	5.00E-05
UCK2	1.64	1.3 - 2.06	3.00E-05
UPB1	0.84	0.76 - 0.93	6.00E-04
USP29	5.99E+42	2.14458024058968e+21 - 1.672272406968e+64	9.00E-05
UTP11L	2.72	1.87 - 3.98	0
VRK2	1.98	1.43 - 2.74	4.00E-05
WASF1	1.53	1.24 - 1.89	9.00E-05
WDHD1	1.95	1.42 - 2.68	4.00E-05
WDR12	1.94	1.32 - 2.87	0.00083
WDR77	1.91	1.39 - 2.62	7.00E-05
XRCC2	2.01	1.41 - 2.85	0.00011
XRCC5	1.82	1.29 - 2.56	0.00061

YARS	2	1.51 - 2.65	0
YBX1	1.97	1.5 - 2.58	0
YEATS2	1.81	1.31 - 2.5	0.00034
YRDC	2.15	1.46 - 3.18	1.00E-04
YTHDF1	2.14	1.42 - 3.21	0.00026
YTHDF2	2.34	1.51 - 3.62	0.00013
YWHAB	1.91	1.36 - 2.67	0.00017
YWHAQ	1.53	1.21 - 1.94	0.00043
ZBTB40	1.89	1.31 - 2.72	0.00068
ZDHHC18	2	1.45 - 2.77	3.00E-05
ZMYM1	1.97	1.35 - 2.88	0.00044
ZMYM4	1.89	1.39 - 2.59	6.00E-05
ZNF131	2.27	1.51 - 3.43	9.00E-05
ZNF239	1.83	1.42 - 2.36	0
ZNF248	2.33	1.45 - 3.76	0.00052
ZNF408	1.84	1.31 - 2.59	0.00044
ZWINT	1.3	1.12 - 1.51	0.00067
